# Fibronectin Regulation by Vitamin C Treatment in Kidneys of Nicotinic Mice Offspring

**DOI:** 10.5812/ircmj.17056

**Published:** 2014-07-05

**Authors:** Hasan Pahang, Mohammad Reza Nikravesh, Mehdi Jalali, Alireza Ebrahimzadeh Bideskan, Peyman Zargari, Ariane Sadr Nabavi

**Affiliations:** 1 Department of Anatomy and Cell Biology, Faculty of Medicine, Mashhad University of Medical Sciences, Mashhad, IR Iran; 2 Department of Human Genetics, Faculty of Medicine, Mashhad University of Medical Sciences, Mashhad, IR Iran

**Keywords:** Vitamin C, Nicotine, Fibronectin, Kidney

## Abstract

**Background:**

Maternal cigarette smoking causes health risks and developmental defects in the offspring. So far, many studies have been conducted to suppress the effects of nicotine. However, the effects of coadministration of vitamin C and nicotine on extracellular matrix have not gained enough attention.

**Objectives:**

This study decided to investigate the effects of vitamin C on fibronectin expression in kidneys of mice offspring, treated with nicotine.

**Materials and Methods:**

Eighteen female pregnant BALB/c mice were selected; six mice in the experimental group 1 (exp 1) received nicotine (3 mg/kg/day), six mice in the experimental group 2 (exp 2) received 3 mg/kg/day nicotine and 9 mg/kg/day vitamin C simultaneously, and six were used as the control group and received 3 mL/kg/day normal saline via intraperitoneal (IP) injection parallel to other groups, since the 6th day of gestation to the end of prenatal period. In the first days of delivery, fibronectin content of neonatal kidneys was studied by immunohistochemistry (IHC) assay and gene expression was studied by the real-time PCR.

**Results:**

IHC results showed that fibronectin reaction significantly increased in proximal convoluted tubules of exp 1 compared with the control offspring; on the other hand, fibronectin reaction decreased in the mice offspring of exp 2. Gene expression results showed that fibronectin expression in the exp 1 offspring significantly increased compared with the control ones and fibronectin expression decreased in the mice offspring of exp 2.

**Conclusions:**

This study revealed that vitamin C could reduce the fibronectin accumulation effects of nicotine on kidney.

## 1. Background

Many mothers who smoke during pregnancy have high morbidity rates of infants (1). Nicotine readily crosses the placenta and its concentrations in serum, amniotic fluid and fetus body are higher in smoking mothers. It gets accumulated in embryonic organs such as kidney (2). Cigarette smoke increased upper airway tracts sensitivity to isoprenaline (3). Furthermore, nicotine exposure in the embryonic period resulted in decreased fibronectin expression in pulmonary parenchyma (4). Maternal nicotine increased collagen reaction in lung parenchyma and caused abnormal bronchogenesis in bronchopulmonary development (5). Nicotine causes developmental defects and weight loss in organs such as kidneys and reduces their angiotensin receptors (6). A recent investigation has demonstrated that nicotine enhances renal diseases and increases the rate of oxidative stress in mice (7). Moreover, it promotes progression of kidney defects. The mechanisms by which nicotine causes progression of renal dysfunction have not been elucidated (8). Cell contact with extracellular matrix has an important role in tissue development and regeneration. Extracellular matrix is composed of glycoprotein, proteinases, and inhibitory structural ligands for cell interaction. Among extracellular matrix components, fibronectin has an important role in cell adhesion, organogenesis, wound healing, blood coagulation, host safety, and metastasis (9). Distribution of fibronectin in normal glomeruli shows that it is present in Bowman's capsule, mesangium, and the parenchyma around the capillary network. Fibronectin is increased significantly in most cases of nephropathy (10). Some investigations have specified that nicotine causes mesangial cells division and cell enlargement via non-neuronal nicotinic acetylcholine receptors. Nicotine exposure induces a slight increase in blood pressure, proteinuria, and increased fibronectin accumulation (11). Some investigations revealed that vitamin C consumption might provide an important role in regeneration of tissue cells. Total ascorbic acid and uric acid reduce H_2_O_2_ synthesis, fibronectin expression, and vascular endothelial growth factor (12). In contrast, in another study, vitamin C up-regulated the expression of type I collagen and fibronectin and induced tissue regeneration (13). Coadministration of vitamin C and lead acetate reduced the severity of degeneration changes and diminished the number of affected organs related to lead acetate alone in rats (14). Another study showed that administration of ascorbic acid in a short period had no considerable effects on tracheal responsiveness in Guinea pigs; however, chronic exposure to ascorbic acid reduced airway responsiveness in these animals (15). Neurodevelopment defects resulting from fetal nicotine significantly decreased with vitamin C consumption (16).

## 2. Objectives

In healthy renal tissues, fibronectin level is conventionally low; but in lesions such as diabetic nephropathy, fibronectin expression increases in basement membranes and extracellular matrix (17). This study decided to assess the relationship between nicotine exposures in prenatal period and fibronectin expression in mice offspring kidneys and determine whether vitamin C is able to change the fibronectin level in renal tissues of mice offspring receiving nicotine during the intrauterine life.

## 3. Materials and Methods

This research was performed in accordance with the rules of the Ethics Committee on Animal Experimentation, Mashhad University of Medical Sciences, Iran. Vaginal plaques in 18 pregnant BALB/c mice, allowed to habituate until embryonic day six, were observed. Afterwards, they were randomly divided into three groups. The Experimental group 1 (exp 1) received 3 mg/kg/day nicotine (Sigma-Aldrich, Saint Louis, USA) via intraperitoneal (IP) injection from the 7th day of gestation to the last day of pregnancy. Exp 2 received 3 mg/kg/day nicotine and 9 mg/kg/day vitamin C (Applichem, Darmstadt, Germany) simultaneously from the 7th day of gestation to the last day of pregnancy. The control group received 3 mL/kg/day normal saline via IP injection from the 7th day of gestation to the last day of pregnancy, similar to other groups. On the first day of delivery, all the infants were anesthetized by chloroform inhalation. Their kidneys were detached and transferred to 10% buffered formalin for immunohistochemical reaction, and RNA stabilization reagent, RNAlater (Qiagen, Hilden, Germany), was added for real-time polymerase chain reaction (RT-PCR).

### 3.1. Immunohistochemical Studies

The right kidneys were removed and transferred to 10% buffered formalin for 24 hours. Immunohistochemical reaction for fibronectin was performed on formalin-rigid sections by an indirect immune peroxidase procedure. Afterwards, the kidney tissues were sectioned at 5 μm thicknesses; they were deparaffinized and rehydrated; then, antigen retrieval was performed in a water bath at 100°C. The sections were blocked with 3% H_2_O_2_ to inhibit the endogenous peroxides activity and transferred to 10% goat serum in phosphate-buffered saline (PBS). Next, they were incubated with specific antifibronectin primary antibody, diluted to 1 in 170 (Abcam, Cambridge, UK), at 4°C overnight, followed by staining with horseradish peroxidase-conjugated secondary antibodies. When the slides were exposed to diaminobenzidine (DAB), brown color appeared. Counterstaining with hematoxylin was performed to clarify the cell nuclei. After dehydrated and stabilized with mounting medium, the stained sections were examined under a light microscope (Olympus BX51, Japan). The intensity of brown color showed the level of fibronectin in renal sections. Image analysis was performed by quantitative scoring methods according to the Table 1 (10):

**Table 1.** The Intensity of Staining

Intensity of Staining	Without Staining	Weak Staining	Moderate Staining	Strong Staining	Very Strong Staining
**Score**	0	1	2	3	4

### 3.2. Real-Time Polymerase Chain Reaction

The left kidneys were removed and gene expressions level was measured by RT-PCR. The kidney samples were homogenized using a laboratory homogenizer (Polytron PT 1200E, Switzerland). Total RNA was extracted from renal fragments of each mouse by RNX-plus (ParsTous, Tehran, Iran), according to the manufacturer’s protocol. The purity of RNA was determined by electrophoresis on agarose gel. Reverse transcription was performed on 3 μg of RNA using a cDNA synthesis kit (ParsTous, Tehran, Iran). RT-PCR was performed on an ABI PRISM® 48-well optical reaction plate (Applied Biosystems StepOne, FosterCity, USA). The RT-PCR mixture contained 1 µL of template (cDNA), 0.2 µM forward primer, 0.2 µM reverse primer, 3.6 µL sterilized water, and 5 µL SYBR green real-time PCR master mix (ParsTous, Tehran, Iran). The glyceraldehyde-3-phosphate dehydrogenase (GAPDH) gene was used as endogenous gene control (18, 19). A relative quantification method was used to compare mRNA expression. Fold changes in mRNA expression were calculated using the 2^-∆∆ct^ equation, where ∆∆CT is the difference between fibronectin and GAPDH genes expressions (20). Each test was performed in triplicates and the expression level was calculated three times. Amplifications for both genes were performed by an optimized protocol (10 minutes at 95°C, 40 repeated cycles of two steps at 95°C for 15 second, 58°C for 30 seconds, 72°C for 30 second, 95°C for 15 seconds, and 55°C for 1 hour).

### 3.3. Oligonucleotide Primers

The sequences of oligonucleotide primers used in RT-PCR were as follows:

Fibronectin, forward primer 5′-taggagaacagtggcagaaag-3′ and fibronectin, reverse primer 5′-ccatcgggactgggttca-3′. GAPDH forward primer 5′-aactcccattcttccacctttg-3′ and GAPDH reverse primer 5′- ctgtagccatattcattgtcataccag-3′. The primers were produced by Oligo Macrogen company (Seoul, Korea).

### 3.4. Statistical Analysis

The Kruskal-Wallis analysis was performed for comparing the nonparametric data between the groups. P < 0.05 was considered statistically significant. Values were offered as means ± SEM.

## 4. Results

**Figure 1.**
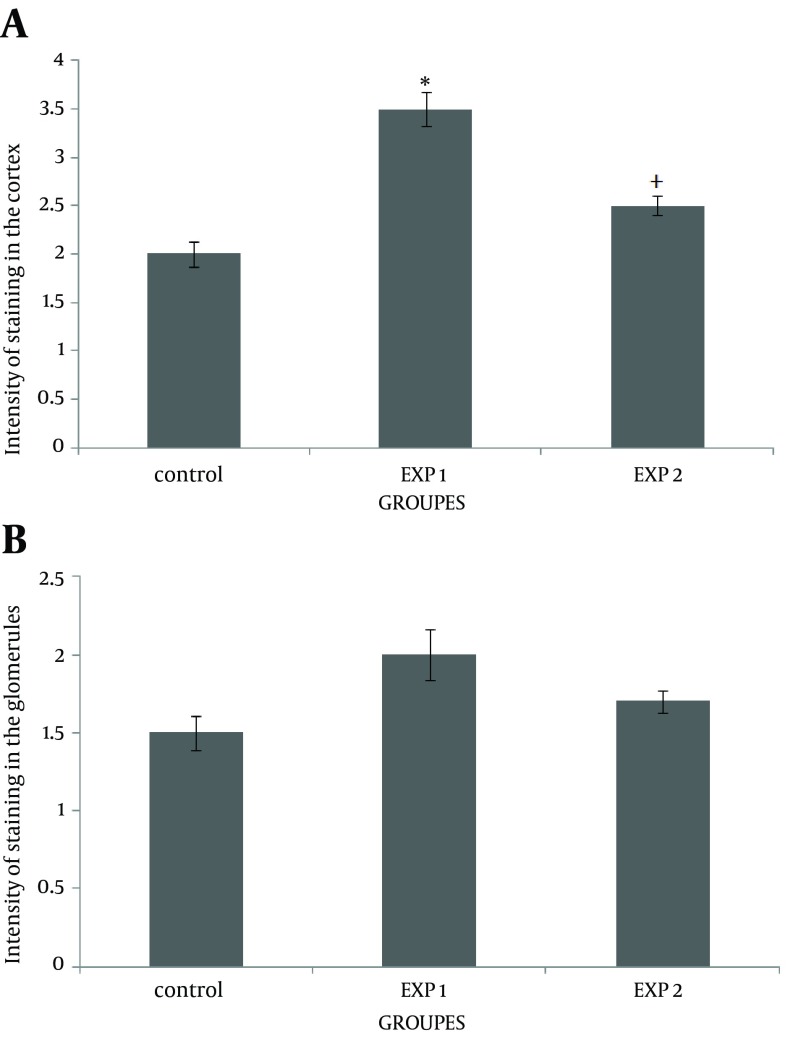
Effects of Nicotine and Nicotine-Vitamin C on Fibronectin Reaction on Renal Cortex and Glomeruli of Mice Offspring Kidneys A) effects of nicotine and nicotine–vitamin C on fibronectin reaction in the renal cortex. ^* ^P < 0.05 versus control and exp 2. ^+ ^P < 0.05 versus exp 1. B) effects of nicotine and nicotine-vitamin C on fibronectin reaction on renal glomeruli. exp 1 received nicotine (3 mg/kg/ day). exp 2 received both nicotine (3 mg/kg/day) and vitamin C (9 mg/kg/day). Values are expressed as means ± SEM of six mice newborns.

**Figure 2.**
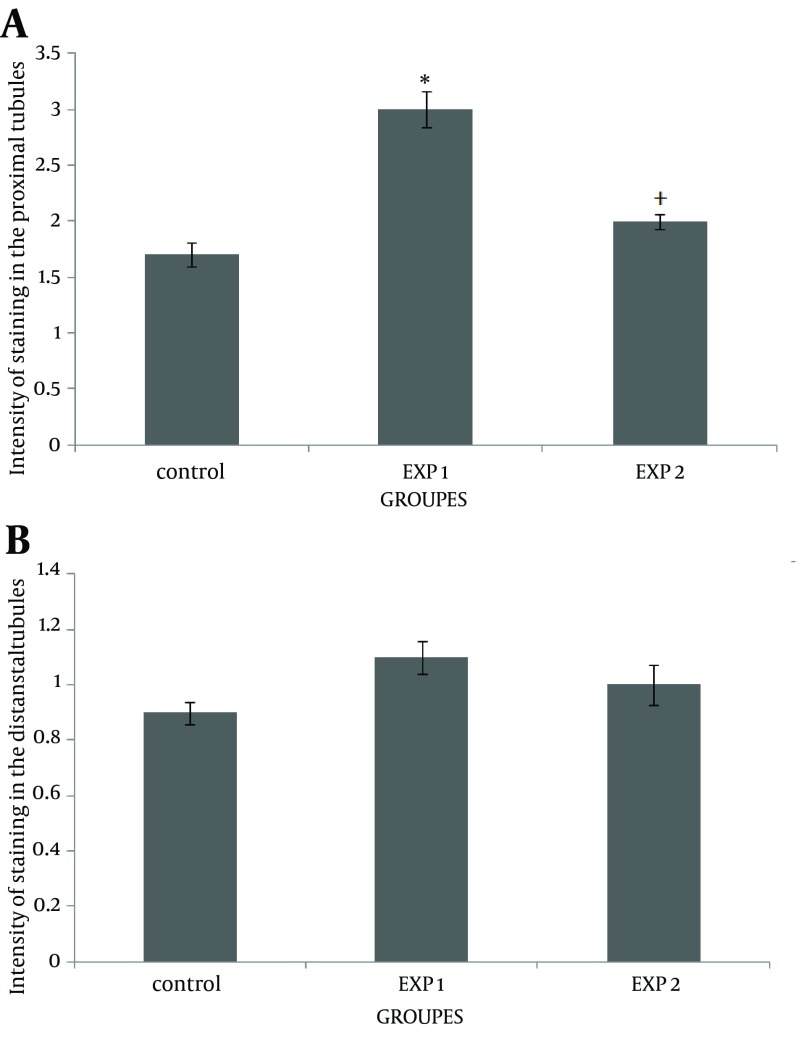
Effects of Nicotine and Nicotine–Vitamin C on Fibronectin Reaction in Proximal and Distal Convoluted Tubules of Mice Offspring Kidneys A) effects of nicotine and nicotine–vitamin C on fibronectin reaction in proximal convoluted tubules. ^* ^P < 0.05 versus control and exp 2. ^+ ^P < 0.05 versus exp 1. B) effects of nicotine and nicotine–vitamin C on fibronectin reaction in distal convoluted tubules. exp 1 received nicotine (3 mg/kg/ day). exp 2 received both nicotine (3 mg/kg/day) and vitamin C (9 mg/kg/day). Values are presented as means ± SEM of six mice newborns.

**Figure 3.**
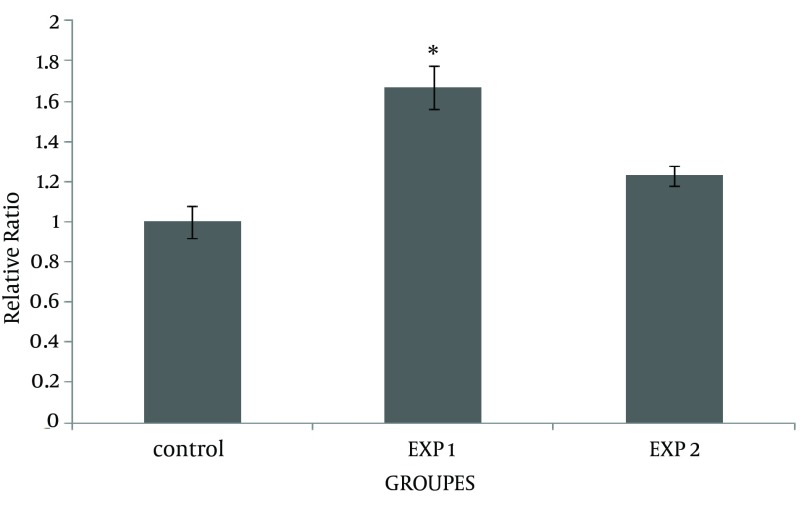
The Results of Real-Time PCR on Fibronectin mRNA Expression in Mice Offspring Kidneys, Received Nicotine and Nicotine–Vitamin C Fibronectin mRNA expression increased in mice offspring kidneys of exp 1. In addition, fibronectin mRNA expression in mice offspring kidneys of exp 2 considerably decreased compared with exp 1. ^* ^P < 0.05 versus control and exp 2. Experiments were repeated three times with similar results and GAPDH was used as an endogenous control.

**Figure 4.**
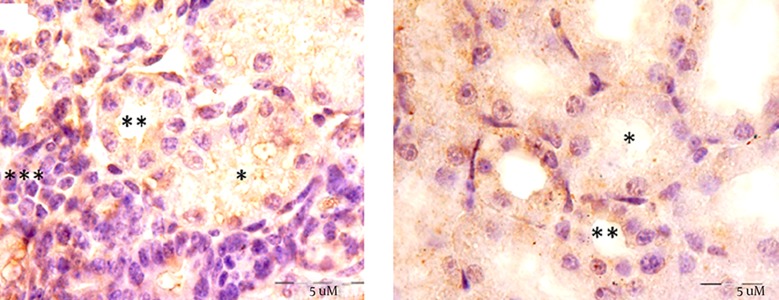
A Transverse Section of Mice Offspring Kidney of the Control Group These images show fibronectin reaction within the proximal^ (*)^, distal convoluted tubule^ (**)^, and glomerulus^ (***)^ of mice offspring kidneys in the control group.

**Figure 5.**
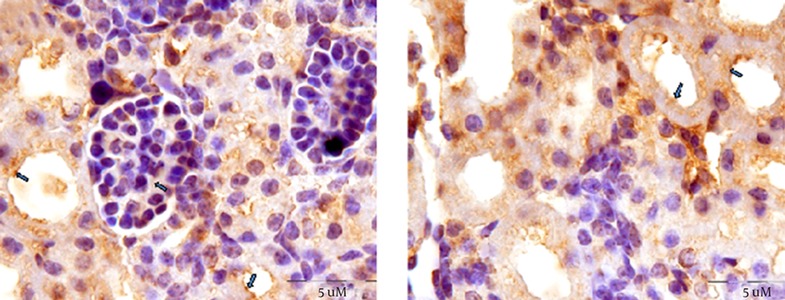
Transverse Section of Mice Offspring Kidney in the Experimental Group 1 In these images, fibronectin reaction was indicated in the proximal convoluted tubule epithelium. Strong reaction also at the apical poles of these epithelial cells as well as extracellular matrix was observed (arrows). In addition, moderate fibronectin reaction was observed in the glomeruli of nicotine exposed mice offspring kidneys.

**Figure 6.**
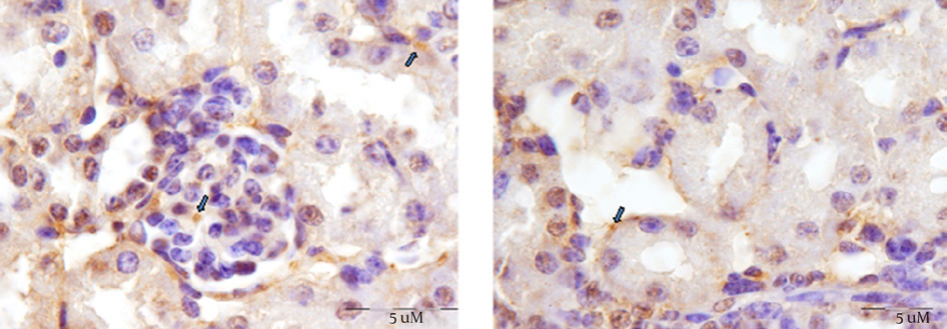
Mice Offspring Kidneys Cross Sections After Nicotine–Vitamin C Treatment in Experimental Group 2 The fibronectin reaction decreased significantly in contrast with the control group. In this group, fibronectin reaction was observed in the basal domain (arrows) of proximal convoluted tubule epithelial cells. Response levels did not change in glomerular extracellular matrix.

This study showed that accumulation of fibronectin increased in the cortex of mice offspring kidneys after nicotine exposure in the prenatal period. Simultaneous injection of nicotine and vitamin C in exp 2 considerably decreased the fibronectin reaction in the renal cortex (Figure 1A). However, fibronectin level did not change in the glomeruli (Figure 1B), compared with the exp 1 (P = 0.047). Data also indicated that fibronectin content was significantly increased in the proximal convoluted tubules of the exp 1, compared with the control group (P = 0.046). Administration of vitamin C decreased the fibronectin level induced by nicotine in the proximal convoluted tubules (Figure 2A). However, nicotine induced fibronectin reaction in the distal convoluted tubules; but it was not significant (Figure 2B). To further confirm the results of this study, mRNA transcription levels of the fibronectin gene were evaluated by RT-PCR (Figure 3). An up-regulation in fibronectin gene expression was observed in exp 1 (0.3 fold change). However, fibronectin gene expression was down-regulated at the end of prenatal period in exp 2 (0.4 fold change). The epithelial cells of proximal convoluted tubules were greatly degenerated in exp 1, in contrast with the control group (Figures 4 and 5). Treatment with vitamin C considerably maintained the extracellular matrix and fibronectin levels in the presence of nicotine. Observation of mice offspring kidney sections in exp 2 showed clear increase in glomerular and urinary space sizes, compared with exp 1 (Figures 5 and 6). In the sections of mice offspring kidneys of exp 2, there was a weighty progress in epithelial cells appearances of proximal convoluted tubules, compared with exp 1 (Figure 6).

## 5. Discussion

Investigations have shown that cell communication with extracellular matrix components has an important role in embryonic morphogenesis and tissue turnover during life and before birth (9). Recent studies have proposed that nicotine causes impaired fertility, type 2 diabetes, obesity, hypertension, and respiratory dysfunction (1). In this study, nicotine significantly increased fibronectin expression in mice offspring kidneys. Therefore; we suggest that nicotine may change the normal function and structure of kidney extracellular matrix. In other words, accumulation of extracellular matrix components causes renal fibrosis and affects kidneys normal function. Fibronectin plays an important role in vascular development and dynamic balance in extracellular matrix (21). A recent investigation showed that nicotine was involved in both pregnancy and kidney disorders (22). Furthermore, nicotine amplifies the degree of renal injury via increasing plasma creatinine level and oxidative stresses such as nitrotyrosine and malondialdehyde (7). Moreover, nicotine induced fibrosis by changing the functions of fibroblasts in organs such as the kidney (23). Depending on the dose and administration time of consuming, nicotine increased metabolic activity of acid phosphatase and cathepsin D in kidney (24). Additionally, fibronectin expression is increased in most types of nephropathy. However, fibronectin decreases in disorders such as mature diabetic nodules, partial scars, and hyalinised glomeruli (10). Matrix metalloprotease A disintegrin and metalloprotease 17 (ADAM17) activation in kidney cortex of diabetic mice led to enhanced activity of NADPH oxidase and increased fibronectin expression. These effects were protected by ADAM17 inhibitor (25). Immunohistochemical findings in the present study revealed that fibronectin reaction in the renal cortex was amplified compared with the control group, confirmed by other investigations. Previous studies indicated that mesangial cell nicotinic ACh receptors in human glomeruli were involved in regulation of Ca^2+^ channel function. Depending on the nicotine dosage, mesangial cells may be involved in overproduction of fibronectin. Reactive oxygen species are made by protein kinase C activation in mesangial cells. NADPH oxidase inhibitors have been able to reduce nicotine-induced renal diseases through mesangial cells proliferation and fibronectin production (8). The present study showed that fibronectin expression increased in kidneys of nicotine-exposed mice offspring. Moreover, increased fibronectin reaction in proximal convoluted tubules of nicotinic mice offspring was observed. Previous studies have revealed that nicotine stimulates renal ischemic injury in mice and increases inflammation process in proximal tubules cells. Presumably, two main factors, namely transcription-3 activator and unphosphorylated signal transducer, are responsible in nicotine-induced acceleration of inflammatory process in extracellular matrix of proximal convoluted tubules epithelial cells (26). The stimulatory effect of nicotine on fibronectin expression may also be mediated through stimulation of immune system macrophages and therefore increase TGFβ1, a famous inducer for fibronectin expression in fibroblasts (27). The important role of vitamin C as an enzyme cofactor, free radical scavenger on hydroxylation reactions, as well as for its antioxidant effects, has recently been noticed (28). Many factors affect uptake of vitamin C during pregnancy. Vitamin C supplementation after 20 weeks of pregnancy significantly reduced the preterm premature rupture of membranes (29). In this study, simultaneous injection of vitamin C with nicotine inhibited the nicotine-induced fibronectin expression. However, addition of vitamin C to the culture medium of human umbilical vein endothelial cells significantly reduced fibronectin production (12). On the other hand, in line with our findings, it has been reported that vitamin C up-regulated the expression of type I collagen and fibronectin in periodontal ligament stem cells (13). Furthermore, low dose of antioxidant improved the thickness of carotid artery after exposure to nicotine, which support the present study results (30). Therefore, the results of the present study suggest that vitamin C decreases the harmful effects of renal fibrosis induced by nicotine. Perhaps, vitamin C inhibits the destructive effects of N-nitroso compounds, produced from reaction of amides or secondary or tertiary amines with nitrite in an acidic background (31). In another study, coadministration of α-tocopherol and ascorbic acid protected the DNA damage resulting from oxidative stress and increased glutathione activity in arsenic-exposed adult rats testicles (32). Glutathione deficiency due to nicotine consumption induced cell death through H_2_O_2_ production and vitamin C was a significant antioxidant in defending cells against oxidative stress. In addition, consumption of vitamin C enhanced intracellular generation of dehydro ascorbic acid exported via the glucose transporters and decreased cellular content of reactive oxygen species (33). Recent investigation showed that interaction of vitamin C with glutathione increased the antioxidant capacity of endothelial cells. These cells contain carrier for oxidized vitamin C and concentrate ascorbic acid with contribution of dehydroascorbic acid reductase. Intracellular vitamin C enhanced glutathione improvement; thus, increased the cells survival potential (34). The present results showed a decrease in the expression of fibronectin in newborns’ kidneys, simultaneously receiving nicotine and vitamin C. Our present data were in general conformity with previous studies suggesting that vitamin C maintained the morphology of cells and down-regulated gene expression (35). The biological properties of vitamin C are not completely understood and more studies are needed to evaluate the role of vitamin C in kidney disorders. However, vitamin C supplementation is recommended for smokers with additional risk factors.
